# Design and Application of an Early Warning and Emergency Response System in the Transboundary Area of the Taihu Lake Basin

**DOI:** 10.3390/ijerph20021340

**Published:** 2023-01-11

**Authors:** Fei He, Qiuying Lai, Jie Ma, Geng Wei, Weixin Li

**Affiliations:** 1 Nanjing Institute of Environmental Sciences, Ministry of Ecology and Environment, Nanjing 210042, China; 2 College of Hydrology and Water Resources, Hohai University, Nanjing 210024, China

**Keywords:** transboundary area of Taihu River Basin, water environmental risk, forecast and early warning, emergency response

## Abstract

The inter-provincial transboundary area of the Taihu Lake Basin is characterized by a complex river network and reciprocating flow. Frequent environmental pollution events in recent years have become a major safety hazard for the water quality in the Taihu Lake Basin. There are few early warning systems for environmental pollution events in China, the ability to simulate risk is insufficient, and systematic research on technology, development, and application is lacking. Thus, water management requirements are not met in the inter-provincial transboundary area of the Taihu Lake Basin. This paper proposes a cross-border risk management plan for pollution sources in the transboundary areas of the Taihu Lake Basin and an early warning and emergency response system for water pollution events using modern information technology. We used this system to assess and classify 2713 risk sources for nitrogen and phosphorus pollution into 5 categories. We simulated the discharge of a pollutant into a tributary and the early warning and emergency response for the transboundary region. The results indicate that the proposed early warning and emergency response system substantially improved the transboundary water environment and lowered the risk of pollution in the Taihu Lake watershed.

## 1. Introduction

The frequent occurrence of environmental pollution incidents in the Taihu Lake Basin in recent years has become a major threat to water quality [1,2]. The occurrence, impact, and risk of environmental pollution events are uncertain [3,4]. China’s ability to deal with sudden environmental pollution events is low, there are few early warning systems, and the ability to simulate risk is insufficient [5,6,7]. In addition, there is a lack of systematic research on technology, development, and application [8]. The transboundary region of the Taihu Lake Basin has faced practical problems.

This study establishes an early warning, risk reduction, and emergency response system for the inter-provincial transboundary area of the Taihu Lake Basin using modern information technology [9]. The key technologies include transboundary risk source risk assessment technology, transboundary water pollution accident early warning technology, transboundary water pollution accident emergency response technology, and transboundary water pollution accident warning technology. Different from the previous studies on risk warning only, this study combines risk assessment, risk warning, three-dimensional visualization simulation, emergency response decision, and accident warning to form a comprehensive system. Information tracking, simulation, analysis, and 3D visualization of Taihu basin can be carried out. The system can be used for risk assessment and early warning, emergency response, and early warning issuance of water pollution incidents.

## 2. Materials and Methods

### 2.1. Overview of the Study Area

The inter-provincial transboundary area of the Taihu Lake Basin refers to the boundary area between Jiangsu and Zhejiang, Jiangsu and Shanghai, Zhejiang and Shanghai, and Zhejiang and Anhui [10,11]. It covers an area of 8280 km^2^ and has a population of 10.69 million, accounting for 1/5 of the entire basin [12,13]. The transboundary area has high economic development and is densely populated [14,15,16,17,18,19]. It has more than 190 large and medium-sized rivers and more than 2000 small rivers, resulting in a complex river network with reciprocating flow [11].

Rapid economic development and resource utilization in the upper and lower reaches and both sides of the transboundary region in the Taihu Lake Basin have caused significant water pollution and environmental management problems in the transboundary region [20,21]. The main problems are as follows: (ⅰ) the scope of the transboundary region has not been clearly defined. The water quality objectives of the upper and lower reaches are not coordinated, and the pollutant flux of the transboundary section is unclear. (ⅱ) The capacity of the early warning and emergency response system to deal with sudden pollution accidents in the transboundary region is weak. (ⅲ) The coordination between administrative units and the ecological compensation mechanism of the river basin in the transboundary area is inadequate. (ⅳ) There is no multi-level or multi-department platform for sharing information on water quality and monitoring at the river basin level, as well as no early warning system for the transboundary region. In addition, a high density of industrial enterprises exists in the transboundary area. Many are high-pollution and high-risk industries, such as the chemical, printing, and dyeing industries [22,23]. Although the situation has improved in recent years after the restructuring of several industries, the problem has not been solved, and there is a hidden danger of sudden cross-border pollution accidents [24].

### 2.2. System Architecture and Key Technologies

#### 2.2.1. System Design Objectives

The objectives of the early warning and emergency response system of the transboundary area of the Taihu Lake Basin are to perform a risk assessment, evaluate water quality, and visualize the risk. The early warning system focuses on the impact of risk events on water quality, water sources, and sensitive aquatic organisms. The spatiotemporal characteristics of environmental pollution events are determined to predict the impact scope, degree, and trend of risks after the occurrence of water pollution accidents. According to the different risk levels, an emergency plan is developed to deal with transboundary water pollution accidents and implement countermeasures. The information is available on a computer network. An established knowledge base of relevant emergency plans and expert advice are used to provide guidance and an emergency response plan that is adjusted and optimized using timely feedback.

#### 2.2.2. System Structure Design

**Figure 1:**
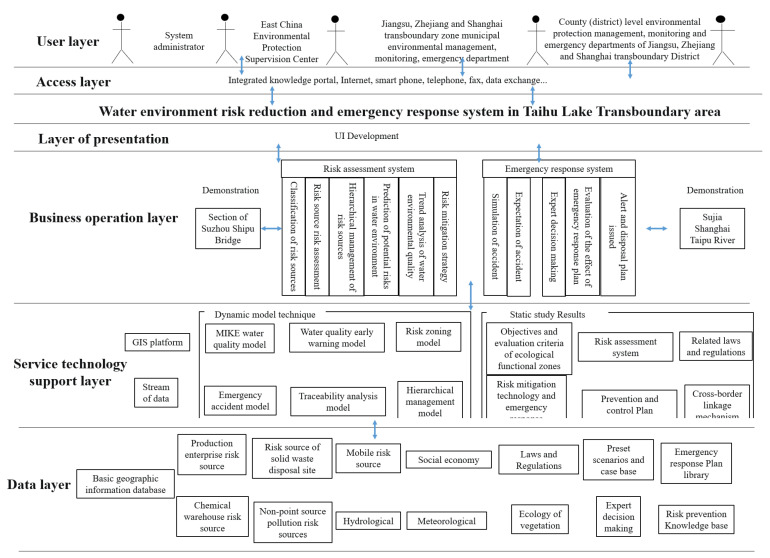
Diagram of the system framework.

In order to be more reasonable and scientific, the system must be safe, reliable, stable, and have a strong comprehensive service capability. The system construction will closely focus on the business needs of the system and carry out scheme design from different perspectives such as data and user business. The specific process of system framework construction is shown in Figure 1.

The scheme design focuses on the overall situation, uses the thinking method of system engineering to grasp the overall situation, and follows the design idea of “platform stability, technology progressiveness, system integrity, structure development, and network adaptability”. The system design is guided by the needs and focuses on science, practicality, progressiveness, scalability, and security, so as to meet the current requirements and to be breakthrough and forward-looking.

The proposed system has three organizational layers: the presentation layer, the business layer, and the business and technical support layer. The data relating to the cross-border water environment are shared using an information exchange system that is accessible to users on the web. The three-layer structure enables data and software sharing, thus facilitating the construction of different user interfaces for different types of subsystems and reducing the difficulty of software development. The hierarchical structure and use of new technology enable the system’s unity and high scalability, which is described as follows:

(i) Presentation layer

The presentation layer consists of the user interface and the result display interface, allowing for scheme editing, model calculation, result display, comparative analysis, evaluation, and information release.

(ii) Business operation layer

The business operation layer is composed of various functional and application modules for the risk assessment and emergency response subsystems. It uses various functional modules for data processing and interaction with the database to perform business transactions requested by users and return the results.

Risk assessment: This module integrates multi-source data information and a risk warning model for water quality, water sources, and sensitive aquatic organisms. It analyzes and evaluates the risk level of the water environment in the transboundary area. It is connected to a GIS interface and is used to visualize the risk in the transboundary area. This module uses Latin hypercube sampling to extract parameters for uncertainty analysis. Sensitivity analysis was performed using global sensitivity analysis and standard rank stepwise regression. Thus, the sensitive parameters of the model, the reasonable range of the parameters, and the contribution rate of the parameters to the uncertainty of the model results are screened and determined. Based on this, the calibration and verification of hydrodynamic, water quality, and ecological key parameters are carried out.

Emergency response and pollutant disposal: This module performs risk warning of water pollution accidents and implements the emergency response plan and pollutant disposal steps according to the risk level.

(iii) Business technical support layer

The business technology support layer includes the core research results, GIS, the MIKE model, and other support modules. The research results include static research results, such as the results of dynamic models and various evaluation systems and standards. The data exchange module performs internal and external data exchange. The GIS platform provides location data and displays other geographic information for other functional modules in the middle layer. The MIKE model provides users with the interface between the scheme and the model. It performs calculations and exports the results of the MIKE model.

#### 2.2.3. Key Technologies

(i) Assessment of risk sources in cross-border areas

The risk sources in the water environment of the transboundary area of the Taihu Lake Basin can be divided into five categories: production enterprises, centralized waste treatment sites, non-point sources, chemical storage sites, and transportation sources [25,26].

**Figure 2:**
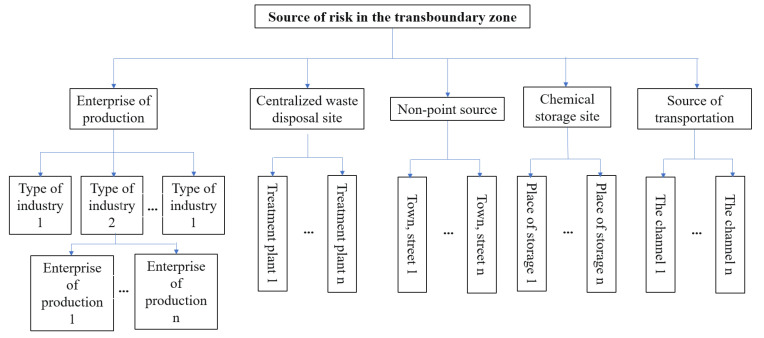
Classification of risk sources in the transboundary area.

The risk sources of production enterprises cover all production industries, with different industry types, enterprise scale and management levels, and risk levels. It is necessary to characterize the risk of all enterprises in the transboundary area. The centralized waste treatment and disposal sites refer to centralized sewage treatment plants. A risk characterization was conducted of all centralized waste treatment and disposal sites in the cross-border area. Non-point source risk sources include surface runoff in the rainy season and agricultural pollution. The risk characterization was performed for each township in the transboundary area. Chemical storage sites refer to the storage sites of eight categories of dangerous chemicals according to the GB13690 standard. The risk analysis for all chemical storage sites was performed. Transportation risks refer to the risks related to waterway transportation in the transboundary area. Each waterway was assessed. Classification of risk sources in the transboundary area is shown in Figure 2.

**Figure 3:**
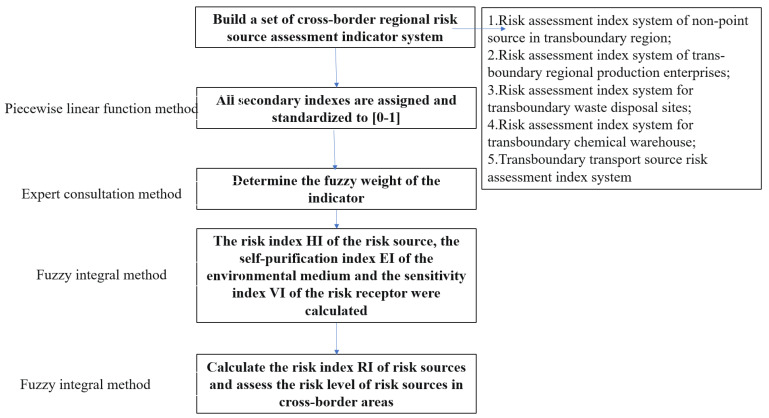
Flow chart of the risk assessment of risk sources in cross-border areas.

The risk level of a risk source is characterized using its risk index (RI). The risk level of the watershed is related to the risk sources, the self-purification of environmental media, and the vulnerability of risk receptors. Therefore, these three aspects were used to construct the risk assessment index system of five types of risk sources. Flow of the risk assessment of risk sources in cross-border areas is shown in Figure 3. We selected 3 primary and 52 secondary indices to evaluate the pollution risk. Assessment index system is shown in Table 1, Table 2, Table 3, Table 4 and Table 5.

The risk assessment index systems for the five types of risk sources all include three first-level indicators: risk source risk, self-purification of environmental media and vulnerability of risk receptors, which are labeled X, Y, and Z, respectively. Each first-level indicator includes a number of second-level indicators of varying numbers labeled x1, x2…; y1, y2…; and z1, z2… According to the potential risk level, each indicator is divided into three risk levels, namely high risk, medium risk, and low risk. An explanation of the meaning of each indicator and how it is calculated is listed at the bottom of each table.

(ii) Water pollution accident warning system in the cross-border area

**Figure 4:**
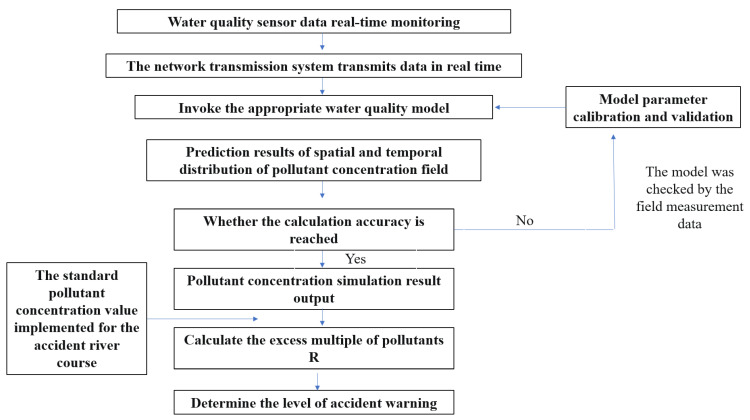
Structure of the accident early warning technology.

A field investigation, GIS and remote sensing analysis, and the results of the cross-border regional risk source assessment were used to evaluate the water quality, aquatic organisms, and risk of pollution. The spatial distribution of the pollutant concentration in the accident pollution scenario (without emergency measures) was simulated. The early warning model was used to simulate the pollutant concentration field and the risk level. Structure of the accident early warning technology is shown in Figure 4.

After the accident, we collected pollution information and called the background model library to determine a suitable water quality model (i.e., dry season model, wet season model, or normal season model). We used real-time sensor data to establish the early warning model, including hydrological and water quality data. The accuracy of the model was analyzed.

(iii) Emergency response for water pollution accidents in the transboundary areas

**Figure 5:**
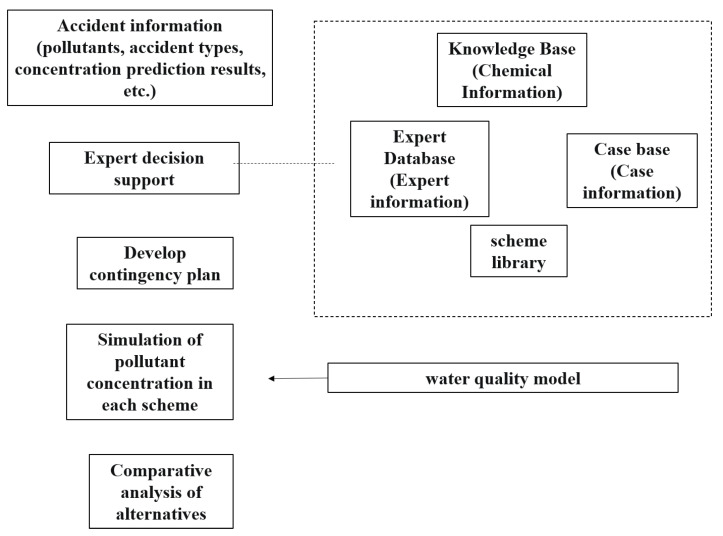
Flowchart of the emergency response system.

The emergency response system should consider the characteristics of the water environment in the transboundary area. Expert decision was used to develop different emergency plans. The performance of the plans was assessed using water quality. The improvement in the water quality was provided to the decision-makers. Flow of the emergency response system is shown in Figure 5.

Due to the complexity and variability of emergencies, the generation and analysis of emergency plans cannot be fully automated, and human intervention and expert opinion are required to make on-site decisions. Therefore, the expert decision-making system is indispensable in accident responses. This system includes four databases: a chemical substance database, an expert database, a case database, and a scheme database.

The expert database information includes an expert directory, unit, research field, and contact information. The chemical repository (knowledge base) information includes common chemical–physical and chemical properties, protection, disposal measures, first-order decay rate constant, etc. The case database includes the accident event, location, event description, pollutant, pollution cause, pollution process, nature of polluted water, whether it is cumulative pollution or sudden pollution, accident disclosure method, accident influence area, accident emergency treatment plan, etc. So far, 661 cases of water pollution accidents have been recorded. Based on research achievements at home and abroad, a set of organizational principles for emergency response based on multi-place and multi-department linkage mechanisms was summarized and formed. The solution library is applicable to emergency treatment technologies and solutions such as rapid interception and transfer of pollution in the transboundary area of Taihu Basin and local rapid disposal, joint control of water quality and quantity, etc.

(iv) Release of alerts of water pollution accidents in transboundary areas

**Figure 6:**
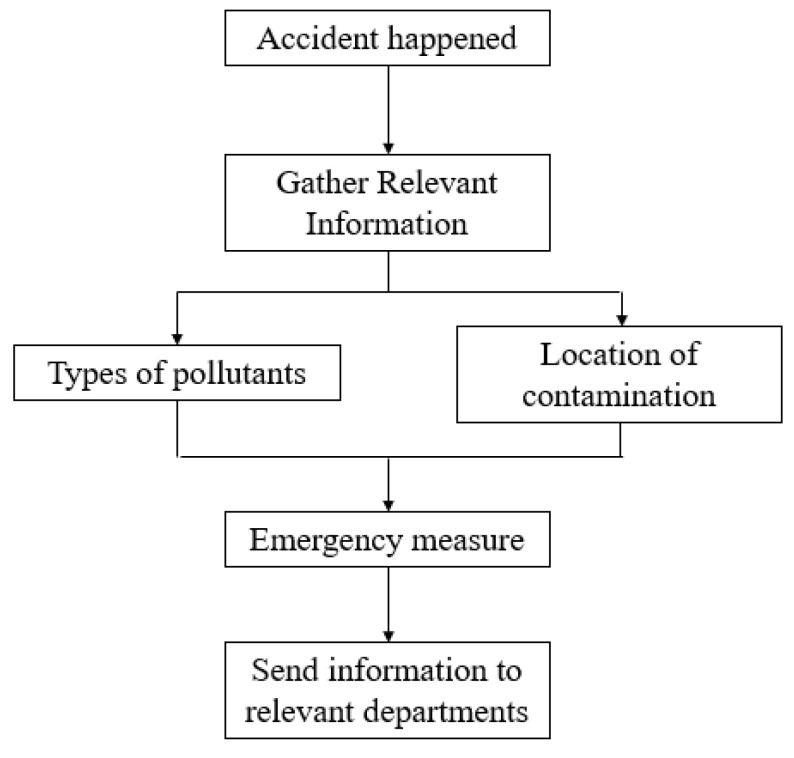
Flow chart of the accident warning system.

After the accident, it is necessary to email an accident early warning report to the different departments. Flow of the accident warning system is shown in Figure 6. The contents of the accident warning report include:

Pollutant: the name, type, nature, and amount of the pollutant.

Accident information: the accident source name (including the responsible person, contact information, and other accident source information) and the cause, location, and time of the accident.

Accident impact and emergency measures: the accident warning level; the length of the pollution zone without emergency measures; the accident impact area; if the accident caused cross-administrative provincial boundary pollution; the highest concentration of pollutants exceeding the water quality standard [27]; the duration when the pollutant concentration exceeded the standard; the affected habitat of rare aquatic organisms or spawning areas for fish and shrimp; the affected species in the water bodies; the effect on drinking water source protection area (if the water source should be closed; the number of people affected by the water source closure; the estimated duration of the water source closure); the emergency treatment method; the regional scope (up to the township level); and departments involved in the multi-regional linkage mechanism. The specific process is shown below.

## 3. Results

### 3.1. Risk Assessment of the Transboundary Area in Taihu Lake Basin

**Figure 7:**
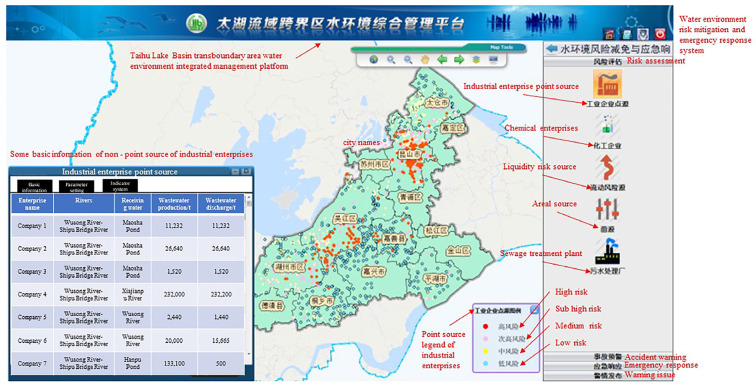
Point source risk assessment results for the industrial enterprises.

**Figure 8:**
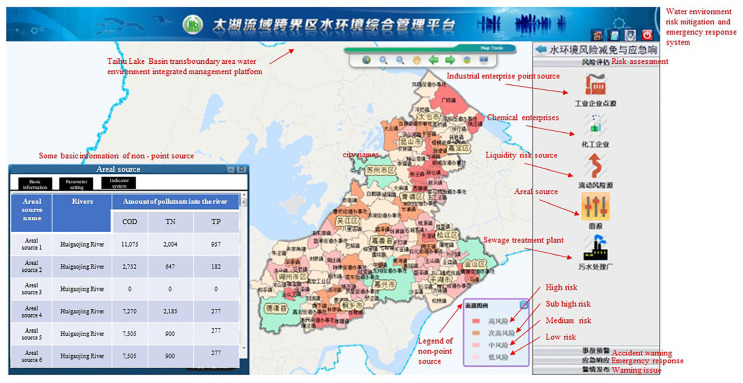
Non-point source risk assessment results.

**Figure 9:**
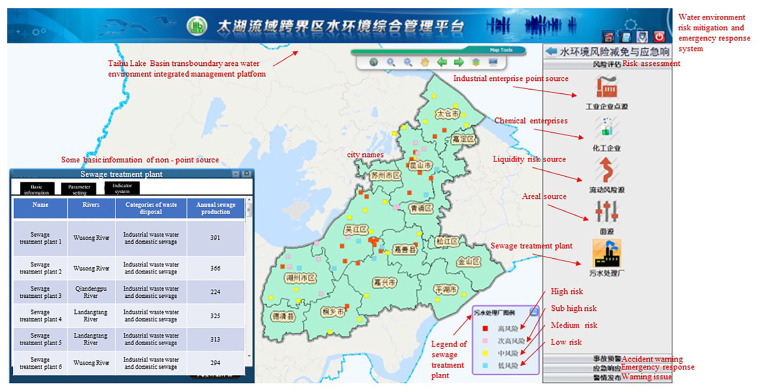
Risk assessment results for the sewage treatment plants.

**Figure 10:**
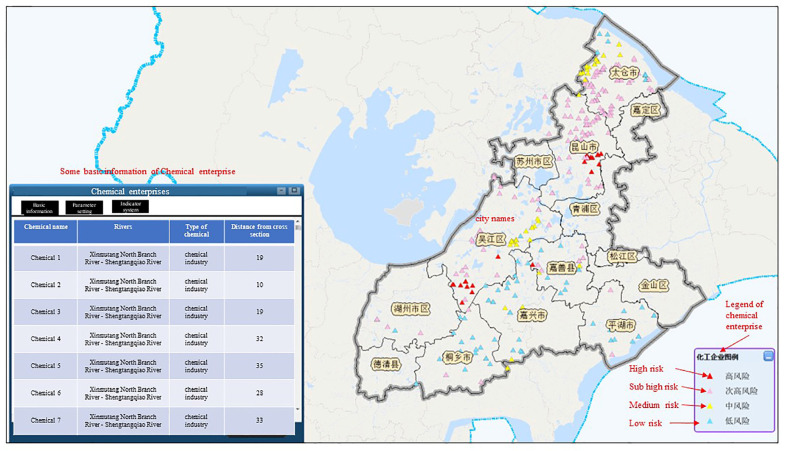
Risk classification of chemical storage sites in the transboundary zones.

**Figure 11:**
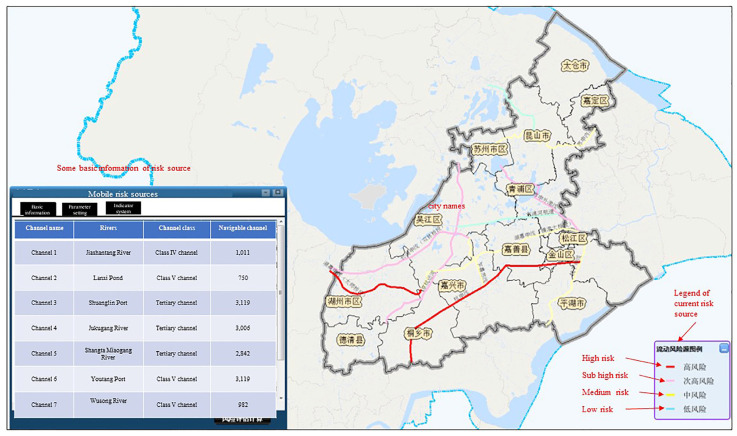
Risk levels of transportation in the cross-border zone.

A total of 2713 risk sources in 5 categories (including production enterprises, non-point sources, mobile sources, chemical warehouses, and waste disposal sites) were evaluated and classified. Risk assessment results is shown in Figure 7, Figure 8, Figure 9, Figure 10 and Figure 11. We removed 320 primary high-risk sources, 517 secondary high-risk sources, and 649 medium-risk sources. The risk prevention and control information of the Taihu Lake Basin in the past 5 years (2016–2020) was systematically analyzed, and a database of risk sources in the transboundary area was established, including 2203 industrial point sources, hydrologic and water quality data on 31 major transboundary rivers, 661 cases of water pollution accidents, more than 200 emergency resource databases, and 130 pieces of expert information. Based on this information, the risk source information database of the transboundary area was established to exchange and share risk source information in the transboundary area of Taihu Lake. This system provides fast and accurate data support for risk source investigation, emergency response, pollution source tracing, and emergency response in the transboundary area. Specific information is shown in Table 6 and Table 7.

### 3.2. Risk Reduction and Emergency Response in the Suhu Taipu River Cross-Border Area

The Taipu River is an important river in the Taihu Lake Basin. It is 57.2 km long and flows through Jiangsu, Zhejiang, and Shanghai, including 40.5 km in Wujiang City in Jiangsu Province, 1.46 km in Jiashan County in Zhejiang Province, and 15.24 km in Shanghai. The Taipu River is an important drinking water source in Jiaxing, Zhejiang Province, and Qingpu District, Shanghai. As a four-level waterway, the Taipu River can be navigated by ships up to 500 tons [28,29,30].

**Figure 12:**
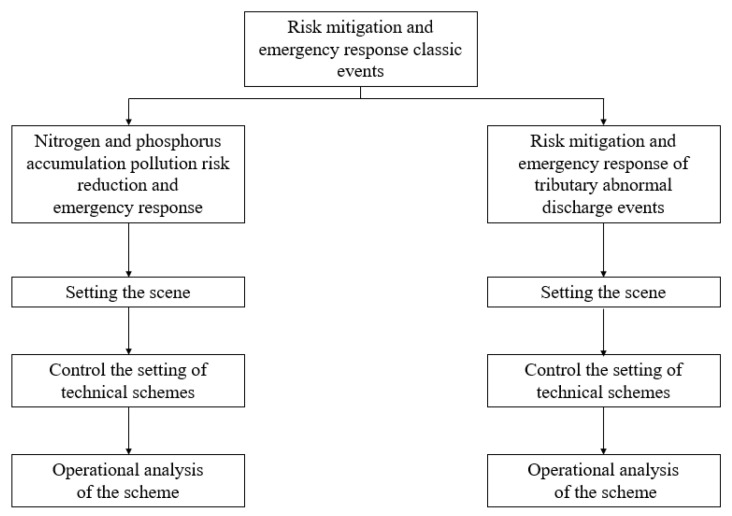
Risk mitigation and emergency response simulation flow chart.

According to the types of risk events in the Taipu River and the distribution of risk sources, two scenarios of risk reduction and emergency response were simulated in the transboundary area of the Taihu River Basin (Taipu River). Specific information is shown in Table 8 and Table 9. Risk mitigation and emergency response simulation flow is shown in Figure 12. The river water quality model in the transboundary area, the basic database of hydrological water quality (including the measured river flow, velocity, water level, flow direction, and other hydrological basic data since January 2013; and river water quality data measured in 13 monitoring points since January 2013: pH, water temperature, dissolved oxygen, COD, BOD5, NH3-N, PO4-P, NO3-N, total nitrogen, total phosphorus, turbidity, TSS, etc.), and the accident source information were used to realize the simulation calculation of emergency accidents and to realize the animation display of the prediction results for the spatial and temporal distribution of the pollutant concentration field in the GIS platform. The details are as follows:

(i) Risk mitigation and emergency response after nitrogen and phosphorus pollution

The following scenario was simulated: During the dry season, ammonia nitrogen at the Suhu boundary of the Taipu River exceeded the class Ⅲ surface water standard due to cumulative pollution.

Scenario details: The monthly variation in the ammonia nitrogen concentration in the Taipu River was high. The data from the Kanazawa section in the last ten years and the measurement obtained by the research group showed that the ammonia nitrogen concentration exceeded the standard in the Taipu River during the first half of the year, and the frequency of exceeding the standard was the highest from January to March. This period is the dry season of the Taipu River, and ammonia nitrogen concentration exceeded the standard by a small amount. Therefore, the excess concentration was attributed to the low flow of the Taipu River in the dry season.

Specific event scenario setting 1: We considered the dry season from January to March. The discharge of water from Taihu Lake was about 50 m^3^/s, and the downstream flow was about 200 m^3^/s. The ammonia concentration in the Suhu transboundary section reached 1.1 mg/L from January to March, slightly exceeding the standard of the class Ⅲ surface water (1 mg/L).

Risk reduction and emergency response: There are two schemes in this event scenario. Scheme 1 was to divert water from Taihu Lake through the Taipu Gate, with an ammonia nitrogen concentration of 0.5 mg/L and diversion flow of 10–50 m^3^/s. Scheme 2 consisted of closing the sluice gates of Wujiang Shijia Port, Wujiang Lanxitang, Wujiang Houchangdang, Jiashan Xihudang, and Jiashan Maxi Lake.

Operability analysis of the scheme: The measured water quality data of the inflow from the upper reaches of the Taipu River showed that the ammonia nitrogen concentration was below 0.5 mg/L, indicating good water quality. In the last three years (2018–2021), the drainage and diversion flow of Taihu Lake through the Taipu Sluice of the Taipu River reached a maximum of 160 m^3^/s from January to March; therefore, the maximum diversion flow for the upper Taipu River in the regulatory scheme of 50 m^3^/s (the total inflow from upstream is 100 m^3^/s) is achievable.

(ii) Risk reduction and emergency response to abnormal discharge events in tributaries

Scenario: The pollutant methylene chloride was discharged into the tributaries of the Taipu River.

Scenario details: In the lower reaches of the Taipu River, the concentration of methylene chloride exceeded the standard. An investigation confirmed that methylene chloride, which is used as a raw material in the chemical industry (Wujiang Jinsui Chemical Co., Ltd. Suzhou, China), was illegally discharged into the Lanxitang tributary. This company manufactures dyes, and the pollution source data showed that the annual wastewater production of the enterprise was 49,000 T. The allowable concentration of methylene chloride in drinking water in China is 0.02 mg/L.

Specific event scenario setting 2: The pollutant methylene chloride was discharged. The industry category was dye manufacturing. The annual wastewater production volume of the enterprise was 49,000 T, and the daily wastewater production was about 145 T. The maximum emission concentration of methylene chloride in the chemical synthesis industry was 0.3 mg/L. If the production wastewater stored by the enterprise for 2 weeks was discharged over 2 h (0.28 m^3^/s) into the Lanxitang tributaries of the Taipingpu River, the concentration of methylene chloride in the production wastewater would be 1000 times (300 mg/L) the standard concentration.

Risk reduction and emergency response: There are three schemes in this event scenario. Scheme 1 was to divert water from Taihu Lake through the Taipu Sluice. The diversion flow was 10–50 m^3^/s, and the water diversion started after the pollutant discharge stopped. Scheme 2 was to close the gate of the tributary of Wujiang Lanxitang after the pollutant discharge stopped. Scheme 3 was to close the sluice gate of the tributary of Wujianglan Xitang and divert water from Taihu Lake through the Taipu Sluice at a flow rate of 10–50 m^3^/s after the pollutant discharge stopped.

Operability analysis of the scheme: In the last three years (2018–2021), the maximum drainage and diversion flow of Taihu Lake via the Taipu Sluice of the Taipu River from January to March was 160 m^3^/s. Therefore, the maximum diversion flow of the upper Taipu River in the regulation scheme was 50 m^3^/s (the total inflow of upstream water was 100 m^3^/s).

(iii) Accident warning

Emergency response: The name, description, location, dangerous goods, and other information on the emergency were recorded. 

**Figure 13:**
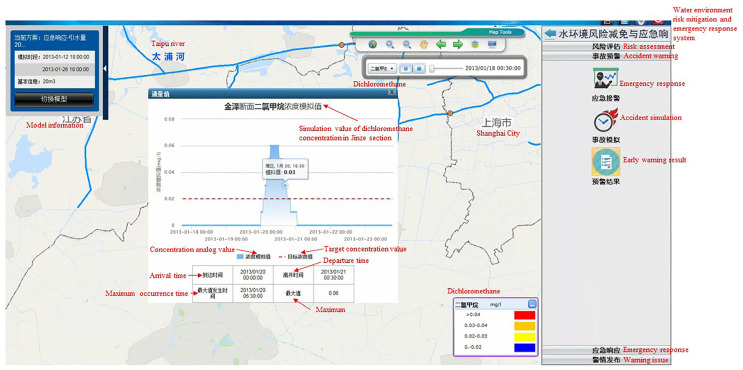
Simulated pollutant concentration values for a pollution accident in the Suhu transboundary area of the Taipu River.

Accident simulation: Accident simulation was used to manage the pollution incident models. Pollution accidents can be created and deleted, and emergency response schemes can be chosen. Boundary information, such as the hydrodynamic boundary and rainfall boundary of the scheme, can be viewed, and the Mike model can be used to simulate emergency responses according to the chosen parameters. As is shown in Figure 13.

(iv) Emergency response

Expert consultation was used to ask experts, in the expert database, questions based on pollutant information. The system displays the expert’s information, including title, technical expertise, and experience in handling pollution accidents. Expert opinions are provided based on the accident simulation model.

**Figure 14:**
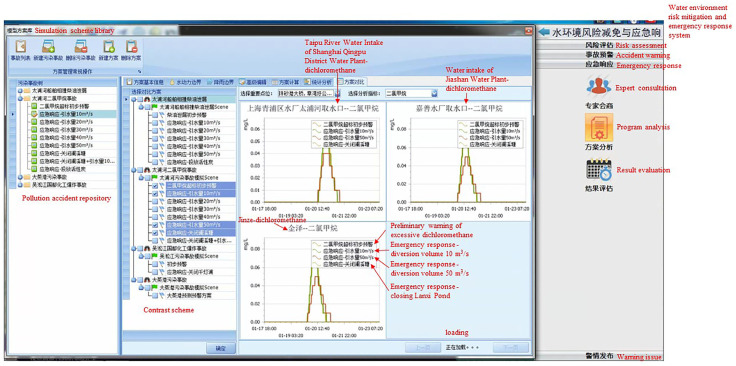
Comparison of emergency response plans for pollution accidents in the Suhu transboundary area of the Taipu River.

**Figure 15:**
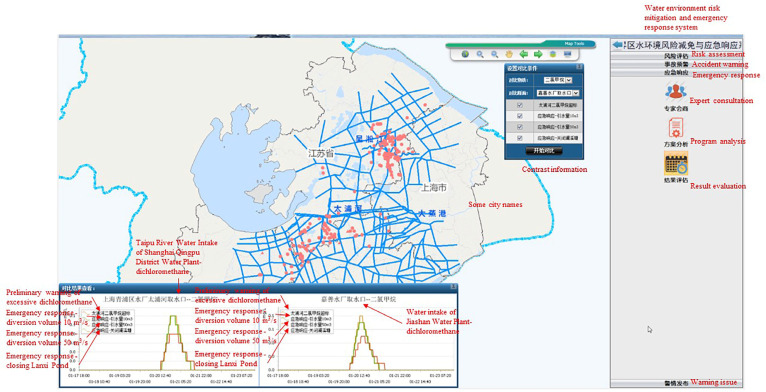
Performance evaluation of emergency plans for the pollution accident in the Suhu transboundary Area of the Taipu River.

Project analysis: Scheme analysis and a comparison of different schemes were conducted to determine the most effective scheme for dealing with pollution accidents. As is shown in Figure 14 and Figure 15.

Publish the results: The simulation results of the early warning and emergency response system are emailed automatically to the relevant departments to provide decision support. This includes the accident and emergency plan, the results of the calculation model, the time when the peak pollutant concentration was reached, the gate control of the tributary, and the water resource.

## 4. Discussion

This demonstration project was located in a watershed boundary area. It is still difficult to obtain and update the measured and online monitoring data of pollution sources and water quality in the study area at this time. This affects the accuracy of model [31,32] predictions to some extent and may lead to bias in management decisions. The operation and maintenance capital budget for the subsequent maintenance and upgrade of the built platform is not clear. In addition, understanding how the uncertainty of the model parameters [33,34], the complexity of the river system, and errors in other input data can lead to accurate prediction in the long term is a great challenge. In the future, the warning system should be further improved by using more complex models, multi-parameter optimization methods, and more intelligent data generation techniques. In line with the current favorable opportunity for various departments to carry out big data construction, the long-term operation and maintenance mechanism for the basic data acquisition and sharing mechanism in major science and technology projects, and the project management business information platform, should be established as soon as possible.

## 5. Conclusions

(i) The proposed early warning and emergency response system consists of a presentation layer, a business layer, and a business and technical support layer. The modular system performs cross-border risk assessment, provides early warning for transboundary water pollution in the Taihu Lake Basin, and implements emergency response plans. We performed simulations of environmental risk prevention and emergency management.
(ii) The proposed system is preliminary. In future applications, additional data should be incorporated to calibrate and validate the model to improve its precision.

## Figures and Tables

**Table 1:** Risk assessment index system for production enterprises in the transboundary zone.

	Level Indicators	Secondary Indicator (Unit)	Grading Criteria and Standardized Scores		
			High Risk (1)	Medium Risk (0.6)	Low Risk (0.2)
T: Risk index (RI)	X: Hazard of risk source (HI)	x_1_: Industry type	Chemical engineering, electroplating, leather, printing and dyeing, paper making	Textile, food processing, etc.	Machinery manufacturing and others
		x_2_: Annual sewage discharge (tons)	500	100	20
		x_3_: Annual COD discharge (tons)	1000	200	20
		x_4_: Annual ammonia nitrogen emissions (tons)	5	2	1
		x_5_: Records of illegal emissions in the last 5 years (times)	3	2	0
	Y: Self-purification of environmental media (EI)	y_1_: Proportion of sewage water quality reaching the standard (%)	10	50	80
		y_2_: Sewage volume flow (m^3^/s)	20	60	100
	Z:Vulnerability of risk receptors (VI)	z_1_: Distance between corporate outfall and cross-boundary section (km)	5	20	30
		z_2_: Distance between enterprise sewage outlet and drinking water source protection area (km)	5	20	30
		z_3_: Presence of rare aquatic species in sewage bodies	3	2	0
		z_4_: Population density (people/km^2^) in the transboundary area	1500	1100	500

**Table 2:** Risk assessment index system for centralized waste treatment and disposal sites in cross-border areas.

	Level Indicators	Secondary Indicator (Unit)	Grading Criteria and Standardized Scores		
			High Risk (1)	Medium Risk (0.6)	Low Risk (0.2)
T: Risk index (RI)	X: Hazard of risk source (HI)	x_1_: Type of waste disposed of	Hazardous waste	Other industrial waste	Other
		x_2_: Annual sewage production (tons)	1000	200	100
		x_3_: Years of operation as a percentage of years of design (%)	80	60	30
		x_4_: Number of pollution accidents in recent 5 years (per)	3	2	0
		x_5_: Number of recorded illegal emissions in the last 5 years (times)	3	2	0
	Y: Self-purification of environmental media (EI)	y_1_: Proportion of sewage water quality reaching the standard (%)	10	50	80
		y_2_: Sewage volume flow (m^3^/s)	20	60	100
	Z: Vulnerability of risk receptors (VI)	z_1_: Distance between site and transboundary section (km)	5	20	30
		z_2_: Distance between site and drinking water source protection area (km)	5	20	30
		z_3_: Rare aquatic species (species) in sewage body	3	2	0
		z_4_: Population density (people/km^2^) in the transboundary area	1500	1100	500

**Table 3:** Risk assessment index system for non-point sources in cross-border regions.

	Level Indicators	Secondary Indicator (Unit)	Grading Criteria and Standardized Scores		
			High Risk (1)	Medium Risk (0.6)	Low Risk (0.2)
T: Risk index (RI)	X: Hazard of risk source (HI)	x_1_: COD inflow from non-point source (kg/d)	8000	4000	1000
		x_2_: non-point source TN into the river (kg/d)	1000	600	200
		x_3_: non-point source TP into the river (kg/d)	250	150	50
	Y: Self-purification of environmental media (EI)	y_1_: Water quality compliance rate in the region (%)	10	50	80
		y_2_: Transboundary water body discharge (m^3^/s)	20	60	100
	Z: Vulnerability of risk receptors (VI)	z_1_: Distance between point source center and transboundary section (km)	5	20	30
		z_2_: Proportion of the length of the protected drinking water source to the length of the transboundary River Channel (%)	2	1.2	0
		z_3_: Number of rare aquatic species in transboundary area (species)	3	2	0
		z_4_: Population density (people/km^2^) in the transboundary area	1500	1100	500

**Table 4:** Risk assessment index system for chemical storage sites in cross-border zones.

	Level Indicators	Secondary Indicator (unit)	Grading Criteria and Standardized Scores		
			High Risk (1)	Medium Risk (0.6)	Low Risk (0.2)
T: Risk index (RI)	X: Hazard of risk source (HI)	x_1_: Chemical type	Highly hazardous	Moderate risk	Mild Risk
		x_2_: Years of operation as a percentage of years of design (%)	80	60	30
		x_3_: Number of leakage accidents in recent 5 years (per)	3	2	0
		x_4_: Location of warehouse	The city	On the outskirts of	rural
	Y: Self-purification of environmental media (EI)	y_1_: Proportion of sewage water quality reaching the standard (%)	10	50	80
		y_2_: Sewage volume flow (m^3^/s)	20	60	100
	Z: Vulnerability of risk receptors (VI)	z_1_: Distance between warehouse and transboundary section (km)	5	20	30
		z_2_: Distance between warehouse and drinking water source protection area (km)	5	20	30
		z_3_: Rare aquatic species (species) in sewage body	3	2	0
		z_4_: Population density (people/km^2^) in the transboundary area	1500	1100	500

**Table 5:** Risk assessment index system for transportation sources in cross-border zones.

	Level Indicators	Secondary Indicator (unit)	Grading Criteria and Standardized Scores		
			High Risk (1)	Medium Risk (0.6)	Low Risk (0.2)
T: Risk index (RI)	X: Hazard of risk source (HI)	x_1_: Route traffic (vessel/day)	2000	1200	200
		x_2_: Volume of dangerous goods transported by sea route (vessel/day)	100	60	20
		x_3_: Number of refueling points on the air route (number)	10	6	2
		x_4_: Number of shipping line loading and unloading terminals	10	6	2
	Y: Self-purification of environmental media (EI)	y_1_: Proportion of navigable water quality reaching the standard (%)	10	50	80
		y_2_: Flow of navigable water bodies (m^3^/s)	20	60	100
	Z: Vulnerability of risk receptors (VI)	z_1_: Nearest distance between cross-boundary section and refueling point (km)	5	20	30
		z_2_: Nearest distance between the cross-boundary section and the pier (km)	5	20	30
		z_3_: Percentage of the protected area of drinking water sources in navigable watercourses in the entire watercourse area (%)	2	1.2	0
		z_4_: Rare aquatic species (species) in sewage body	3	2	0
		z_5_: Population density (people/km^2^) in the transboundary area	1500	1100	500

**Table 6:** Composition of risk sources in the transboundary regions.

Level of Risk Source	High-Risk	Second-Highest Risk	Medium Risk
Quantity	320	517	649

**Table 7:** Transboundary region risk source basic information database.

Information Database Composition	Industrial Point Source	River Information	Accident Case Information	Emergency Resource Baseinformation	Expert’s Information
Quantity	2203	31	661	200	130

**Table 8:** Risk reduction and emergency response for scenario 1.

Scenario	Control Measures	Details
The ammonia nitrogen concentration at the Suhu boundary of the Taipu River exceeds the standard of class Ⅲ surface water in the dry season	Scheme 1: Water is diverted from Taihu Lake via the Taipu Sluice	1. The water diversion flow is 10 m^3^/s (the inflow from upstream is 60 mL^3^/s)
		2. The diversion water flow is 20 m^3^/s (the inflow from upstream is 70 m^3^/s)
		3. Water is diverted at a flow rate of 30 m^3^/s (the inflow from upstream is 80 m^3^/s)
		4. The diversion flow rate is 40 m^3^/s (the inflow from upstream is 90 m^3^/s)
		5. The diversion flow rate is 50 m^3^/s (the inflow from upstream is 100 m^3^/s)
	Scheme 2: Close tributary gates during the dry period	6. Close the Shijia Harbor tributary
		7. Close the Lanxitang tributary
		8. Close the Post Changdang tributary
		9. Close the Xihudang branch
		10. Close the branch of Maxie Lake

**Table 9:** Risk reduction and emergency response technical scheme setting for event scenario 2.

Scenario	Control Measures	Details
Discharge of methylene chloride into the Pingwang Tributary of the Taipu River	Plan 1: Divert water from Taihu Lake via the Taipu Gate	1. Divert water at a flow rate of 10 m^3^/s
		2. Divert water at a flow rate of 20 m^3^/s
		3. Divert water at a flow rate of 30 m^3^/s
		4. Divert water at a flow rate of 40 m^3^/s
		5. Divert water at a flow rate of 50 m^3^/s
	Option 2: Close the Lanxitang tributary	6. Close the Lanxitang tributary
	Plan 3: Divert water from Taihu Lake and close the Lanxitang tributary	7. Close the gate and divert water at a flow rate of 10 m^3^/s
		8. Close the gate and divert water at a flow rate of 20 m^3^/s
		9. Close the gate and divert water at a flow rate of 30 m^3^/s
		10. Close the gate and divert water at a flow rate of 40 m^3^/s
		11. Lock divert water at a flow rate of 50 m^3^/s

## Data Availability

The data used during the study appear in the submitted article.
